# Supplementary material for: Perceived Parental Attitudes Are Indirectly Associated with Consumption of Junk Foods and Sugar-Sweetened Beverages among Chinese Adolescents through Home Food Environment and Autonomous Motivation: A Path Analysis
Source: Nutrients. 2021 Sep 27;13(10):3403. doi: 10.3390/nu13103403 (PMC8541436; doi:10.3390/nu13103403)
Supplement: Supplementary file 1 [file nutrients-13-03403-s001.zip › nutrients-1383261-supplementary.pdf]

Table S1. Measures of Junk Food/Sugar-Sweetened Beverage Consumption, Perceived Parental Attitudes, Home Food Environment and Autonomous Motivation.

	Items	Response options	Scoring
JF consumption	How many times did you eat the following foods during the past 7 days?<br>a. Fried potatoes.<br>b. Candy or chocolate.<br>c. Cookies, cakes, cupcakes, doughnuts, brownies, pop-tarts<br>d. Ice cream or other frozen desserts.<br>e. Regular potato chips, corn chips or cheese puffs<br>f. Sugary cereals.	Never = 0, 1 – 3 times in the past 7 days = 0.3, 4 – 6 times in the past 7 days = 0.7, 1 time per day = 1, 2 times per day = 2, 3 or more times per day = 3.	Total score
SSB consumption	How many times did you consume the following drinks during the past 7 days?<br>a. Sweetened fruit drinks and teas.<br>b. Regular soda.<br>c. Energy drinks	Never = 0, 1 – 3 times in the past 7 days = 0.3, 4 – 6 times in the past 7 days = 0.7, 1 time per day = 1, 2 times per day = 2, 3 or more times per day = 3.	Total score
Perceived parental attitudes (“PARENT” means the adult who takes care of you. It could be your birth mother or father or your adopted mother or father. It could also be your guardian, and adult relative or an adult who isn’t related to you.)	What your parent(s) say or do when it comes to eating JFs or drinking SSBs?<br>a. If I’ve had a bad day, my parent(s) let me have JFs or SSBs to make me feel better.<br>b. My parent(s) don’t buy a lot of JFs or SSBs for me.<br>c. My parent(s) try to avoid eating JFs or drinking SSBs when I’m around.<br>d. My parent(s) have to make sure that I don’t eat too much JFs or drink too much SSBs.	Strongly disagree = 5, Somewhat disagree = 4, Neither disagree nor agree = 3, Somewhat agree = 2, Strongly agree = 1.<br><br>Strongly disagree = 1, Somewhat disagree = 2, Neither disagree nor agree = 3, Somewhat agree = 4, Strongly agree = 5.	Average score
JF availability in home environment	How often are the following foods available in your home?<br>a. Sweets like candy, cookies, cake, ice cream, etc.<br>b. Regular potato chips, corn chips or cheese puffs	Never = 1, Rarely = 2, Sometimes = 3, Often = 4, Always = 5.	Average score
SSB availability in home environment	How often are the following drinks available in your home?<br>a. Sugary drinks like regular soda, sports drinks, fruit drinks, sweetened teas and other drinks with added sugar	Never = 1, Rarely = 2, Sometimes = 3, Often = 4, Always = 5.	
Autonomous motivation	There are lots of reasons why people might limit the amount of JFs and SSBs they have. Please	Strongly disagree = 1, Somewhat disagree = 2, Neither disagree nor agree =	Average score

	select how much you disagree or agree with?<br>a. I have thought about it and decided that I want to limit JFs and SSBs.<br>b. It's an important thing for me to do.	3, Somewhat agree = 4, Strongly agree = 5.	

JF, junk food; SSB, sugar-sweetened beverage.
